# Lower Abdominal vs. Lateral Thigh Perforator Flaps in Microsurgical Sarcoma Reconstruction: The Aesthetics of Donor Site Matters

**DOI:** 10.3390/jcm13123622

**Published:** 2024-06-20

**Authors:** Beniamino Brunetti, Rosa Salzillo, Riccardo De Bernardis, Valeria Petrucci, Matteo Pazzaglia, Chiara Camilloni, Alessandra Putti, Marco Morelli Coppola, Stefania Tenna, Paolo Persichetti

**Affiliations:** Department of Plastic, Reconstructive and Aesthetic Surgery, Fondazione Policlinico Universitario Campus Bio-Medico di Roma, 00128 Rome, Italy; b.brunetti@policlinicocampus.it (B.B.); riccardo.debernardis@unicampus.it (R.D.B.); v.petrucci@unicampus.it (V.P.); matteo.pazzaglia@unicampus.it (M.P.); chiara.camilloni@unicampus.it (C.C.); alessandra.putti@unicampus.it (A.P.); m.morellicoppola@policlinicocampus.it (M.M.C.); s.tenna@policlinicocampus.it (S.T.); p.persichetti@policlinicocampus.it (P.P.)

**Keywords:** microsurgery, ALT, SCIP, DIEP, reconstructive, aesthetic, sarcoma, SCAR-Q

## Abstract

**Introduction:** Sarcoma resection often leaves patients with big defects only amenable through microsurgical reconstruction. In such cases, it is hard for the surgeon to uphold low donor-site morbidity with an aesthetic result. The purpose of this study was to investigate the clinical outcome and the patient’s perception regarding the donor site in a cohort of patients undergoing microsurgical reconstruction with lateral thigh and lower abdominal perforator flaps. **Methods:** A retrospective evaluation of all patients who underwent sarcoma reconstruction with flaps harvested from the lower abdominal region (deep inferior epigastric artery perforator flap, superficial circumflex iliac artery perforator flap) or lateral thigh region (anterolateral thigh perforator flap and its variations) was performed. Only patients with defects greater than 100 cm^2^ were included. Patient demographics and operative variables were recorded, together with complications. Patient satisfaction and quality of life with the donor site were registered using the SCAR-Q questionnaire, which was administered at least six months post-operatively. **Results:** Eighteen anterolateral thigh (ALT) perforator flaps and twenty-two deep inferior epigastric artery perforator (DIEP) and superficial circumflex iliac artery perforator (SCIP) flap procedures were performed. The two groups were homogeneous for major post-operative complications (*p* > 0.999). Patient satisfaction with the donor site measured using the SCAR-Q questionnaire showed significantly higher scores in the DIEP/SCIP group when compared with the thigh group (*p* < 0.001), indicating a superiority of the lower abdominal area as an aesthetic donor site. **Conclusions:** The DIEP and SCIP flaps are a versatile option for reconstructing large soft-tissue defects following sarcoma resection. Therefore, flaps harvested from the lower abdomen yield a higher patient satisfaction with the donor site, which is a feature worth considering when planning a reconstructive procedure.

## 1. Introduction

The conventional surgical approach for sarcomas involves a wide excision aimed at achieving negative margins (R0 status, indicating the absence of residual microscopic disease). To ensure R0 resection, the incision should traverse grossly normal tissue planes uncontaminated by the tumor. Wide excision results in huge soft-tissue defects, eventually leading to a significant impairment in function and body appearance. In the last 20 years, a paradigm shift in the surgical management of soft-tissue sarcomas (STSs) has been observed, with the introduction in clinical practice of advanced reconstructive procedures, allowing us to reduce the need for amputation and to preserve as much form and function as possible [1,2].

Local and locoregional flaps are often not an option, particularly for larger defects or in cases of previous radiotherapy, which causes tissue fibrosis and yields a higher risk of complications [3]. Microsurgical flaps offer the opportunity to reconstruct large and deep soft-tissue defects bringing healthy and well-vascularized tissue within a challenging environment, hence enabling the acceleration of healing and lowering the occurrence of wound complications [4]. This mitigates the risk of delays in commencing or continuing life-saving oncological therapies.

**Figure 1:**
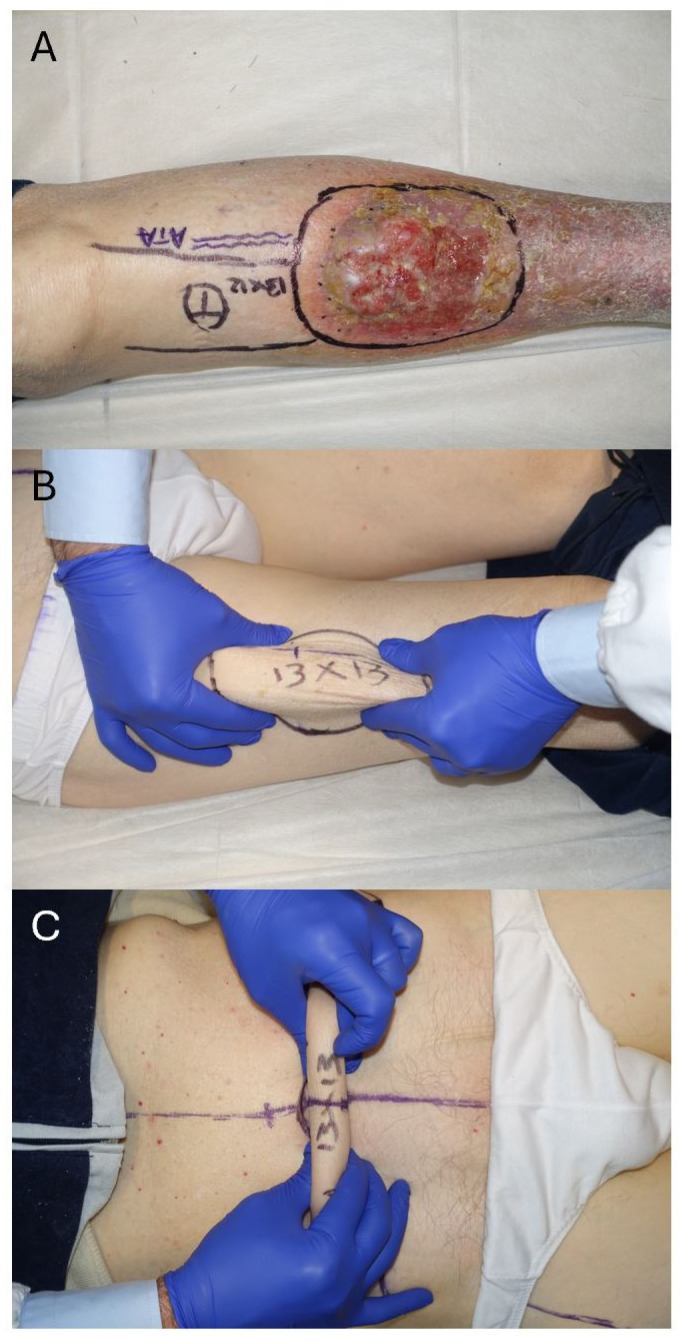
(**A**) Flap selection for a lower limb reconstruction with an extensive defect expected (13 × 13 cm). (**B**) A free ALT flap is one of the most common reconstructive options employed in sarcoma patients, but its donor site will require skin grafting for defects exceeding 8–9 cm in width. (**C**) The lower abdominal region (DIEP flap) allows the harvesting of larger flaps (10–17 cm in width) while achieving primary closure of the donor site in an aesthetic abdominoplasty fashion.

The body’s repertoire of viable flaps diminishes significantly when faced with the need for exceptionally large reconstructions. Among the array of available options to treat extensive defects, several significant approaches stand out. The most common solution employed is the latissimus dorsi flap, either muscular and covered with a skin graft or myocutaneous harvested as a “kiss” flap [5,6,7], which provides a reliable amount of tissue for coverage, albeit with limitations regarding donor site morbidity and aesthetic outcomes. Another workhorse to reconstruct medium to large-sized defects after sarcoma resection (Figure 1A) is the anterolateral thigh (ALT) flap, which, despite its widespread use, unfortunately, may entail higher morbidity at the donor site for flaps exceeding the possibility of primary closure (Figure 1B), eventually leading to less aesthetically pleasing outcomes.

Conversely, advancements in microsurgical techniques have popularized alternatives such as the deep inferior epigastric perforator (DIEP) flap, initially acclaimed for breast reconstruction but now recognized for its versatility in addressing various tissue deficits (Figure 1C). Similarly, the superficial circumflex iliac artery (SCIP) flap, frequently described as a pedicled flap, has emerged as a viable option as a free flap, offering distinct advantages in certain clinical scenarios.

Consideration of donor site morbidity is paramount when undertaking extensive reconstructions required in sarcoma surgery. It is essential to also consider the impact of these procedures on patients utilizing validated assessment tools such as Patient-Reported Outcome Measures (PROMs) [8]. Specifically, the choice of the ideal flap should also be guided by the flap whose donor site restoration implies good patient satisfaction, which can be investigated using the SCAR-Q questionnaire [9,10].

This paper reports our institutional experience in treating extensive soft-tissue defects consequent to sarcoma resection using free perforator flaps harvested from the lower abdomen and the lateral thigh regions. These two groups of patients were compared in terms of clinical results and patient satisfaction with the donor site using the SCAR-Q questionnaire. By creating a decision-making schema, we sought to streamline the process of flap selection, taking into account not only the reconstructive needs of the recipient site but also the aesthetic impact that surgery may have on the patient’s overall appearance and quality of life according to the different donor sites.

## 2. Materials and Methods

A retrospective evaluation of all patients who underwent oncological sarcoma resection and microsurgical reconstruction with either lower abdominal perforator flaps (DIEP, SCIP) or lateral thigh perforator flaps (ALT and its perforator variations, such as AMT and TFL) at Fondazione Policlinico Universitario Campus Bio-Medico of Rome (Rome, Italy) between 2019 and 2023 was performed. All cases were scheduled for pre-operative evaluation at the institutional multidisciplinary team (MDT) meetings held weekly for patients affected by soft-tissue sarcomas (STS).

The inclusion criteria were as follows: patients with diagnoses of STS requiring malignancy-wide excision and immediate reconstruction with a flap; defects greater than 100 cm^2^; flaps wide at least 8 cm; follow-up ≥ 6 months.

Defects amenable to primary closure and/or skin grafting, defects smaller than 100 cm^2^, and patients with a follow-up < 6 months were excluded from this study.

The collected information included patients’ baseline characteristics and morbidities, data about the location and size of the defects, and details about the surgical procedure (duration, flap type and dimensions, source vessels, and recipient vessels for free flaps).

After a minimum of 6 months post-operatively, patients were administered the SCAR-Q questionnaire investigating their satisfaction and quality of life with the donor site.

The normality of the study sample was checked using a Shapiro–Wilk test. Variables following a Gaussian distribution in the two groups were compared using Student’s t-test with 95% confidence intervals and two-tailed *p*-values, whereas variables not following a Gaussian distribution in the two groups were compared using a Mann–Whitney U test with 95% confidence intervals and two-tailed *p*-values. Nominal variables were analyzed using Fisher’s exact test with two-tailed *p*-values. Prism GraphPad (version 9.3.1, San Diego, California) and Microsoft Excel (Microsoft Corp., Redmond, Washington) were used for statistical analysis.

## 3. Results

Forty microsurgical reconstructions were evaluated in this study. The defects were located in the lower extremity, trunk, upper extremity, and head and neck region in 14, 13, 11, and 2 cases, respectively. Fifteen ALT flaps, two TFL flaps, one AMT flap, seven SCIP flaps, and 15 DIEP flaps were performed. Bipedicled DIEP flaps were used in five cases. The mean age, BMI, defect and flap dimensions, donor site complications, and need for a skin graft for donor site closure are listed in Table 1.

No differences were observed between the abdomen donor site group and the thigh donor site group concerning patients’ age (*p* = 0.994) and BMI (*p* = 0.355). The mean defect size was 292 cm^2^ (range 150–540 cm^2^) in the abdominal flap group and 247 cm^2^ (range 108–600 cm^2^) in the thigh flap group (*p* = 0.123). The mean flap sizes were 282 cm^2^ (range 150–540 cm^2^) and 264 cm^2^ (range 165–600 cm^2^) in the abdominal and thigh flap groups, respectively (*p* = 0.607).

The mean operative times were 655.2 min (range 330–918 min) and 527 min (range 318–706 min) in the abdominal and thigh flap groups, respectively (*p* = 0.003).

The donor site required a skin graft for closure in 10 cases belonging to the thigh flap group and none belonging to the abdominal flap group (*p* = 0.002).

Two cases of flap loss were registered (one ALT and one SCIP), which were reoperated with a free LD flap and a pedicled LICAP flap, respectively. Complications at the recipient site were not significantly different between the two groups (*p* > 0.999), while donor site complications were significantly higher in the thigh flap group (*p* = 0.017).

The mean healing times were 31.3 days and 80 days in the abdominal and thigh flap groups, respectively, equating to a statistically significant difference with *p* = 0.0005 (Table 1).

Patient satisfaction with the donor site measured using the SCAR-Q questionnaire showed significantly higher scores in the abdominal flap group when compared with the thigh flap group in every domain: appearance scale (*p* < 0.001), symptoms scale (*p* < 0.001), and psychosocial well-being (*p* = 0.007), indicating the superiority of the lower abdominal area as an aesthetic donor site (Table 2).

## 4. Discussion

Choosing the appropriate free flap is essential to restore both function and aesthetics effectively after extensive sarcoma resection. This is particularly significant for patients who may face significant changes in body image post-surgery. Thus, utilizing flaps harvested from donor sites that can be closed in an aesthetically pleasing manner becomes paramount.

**Figure 2:**
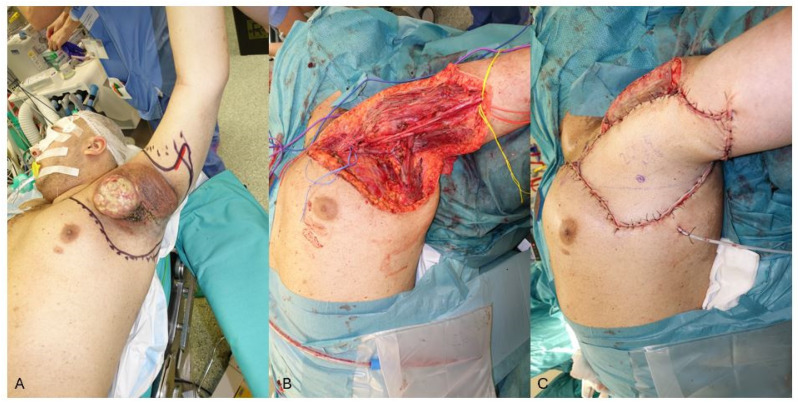
(**A**) A young male patient affected by NF-1 presenting with a huge malignant peripheral nerve sheet tumor (MPNST) involving the brachial plexus and proximal upper arm. (**B**) Intra-operative view of the extensive defect with exposure of the major neuro-vascular structures. (**C**) The reconstruction was performed with a 26 × 15 cm free ALT flap anastomosed to subclavian vessels.

In our initial experience with sarcoma patients, we addressed such complex defects with workhorse flaps such as the ALT flap [11], because of its relatively constant anatomy, reliable vascularization, long vascular pedicle, and extreme variability in design due to the abundance of perforators and possibility to vary the flap thickness according to the defect. This flap can be raised as a thin flap, which is useful for the reconstruction of regions in which the restoration of the normal range of motion is paramount, like the axilla (Figure 2) or the foot [12], or, in cases where the is a need to fill a deep dead space. Moreover, as recently described [13], the ALT flap skin island can be combined with the harvest of the vastus lateralis or rectus femoris muscles to provide functional reconstruction in cases of quadriceps or calf compartments resections [14]. Generally, the maximum width for primary closure of the ALT donor site is 8 to 10 cm [15,16], which is achievable only in cases of significant skin laxity.

**Figure 3:**
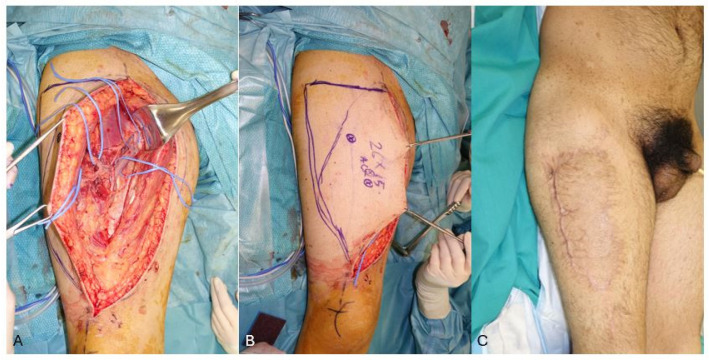
(**A**,**B**): Intra-operative view of the ALT flap based on 2 septo-cutaneous perforators. The donor site was closed with a split-thickness skin graft. (**C**): Hypertrophic scars with a poor aesthetic result at the ALT donor site, 3 months post-operatively.

However, the morbidity of the donor site will increase since primary closure will not be possible, requiring a skin graft and thus worsening the aesthetic and functional impact (Figure 3). This was reflected in our series, where 55% of the ALT donor sites were skin grafted (Table 1), delaying in a statistically significant manner the final healing and leading to poorer aesthetic outcomes, as shown by the SCAR-Q results (Table 2).

**Figure 4:**
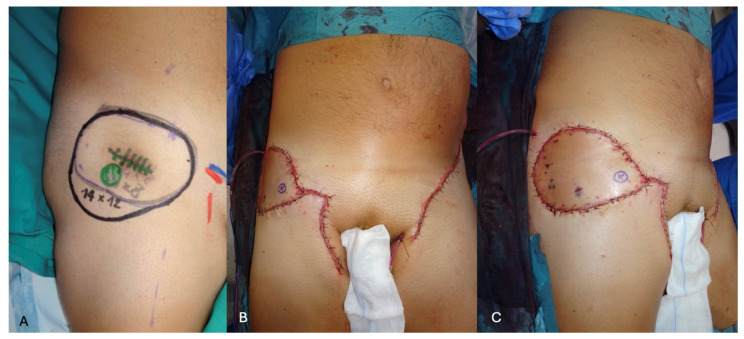
(**A**) Recurrent liposarcoma after irradiation in the right flank, with the 14 × 12 cm planned resection. (**B**,**C**) Intra-operative view of the transplanted SCIP flap after revascularization. The left superficial circumflex iliac artery perforator was anastomosed to its contralateral counterpart in a perforator-to-perforator fashion. The flap vein (SCIV) was of a bigger caliber and required anastomosis to a saphenous vein branch of adequate size.

**Figure 5:**
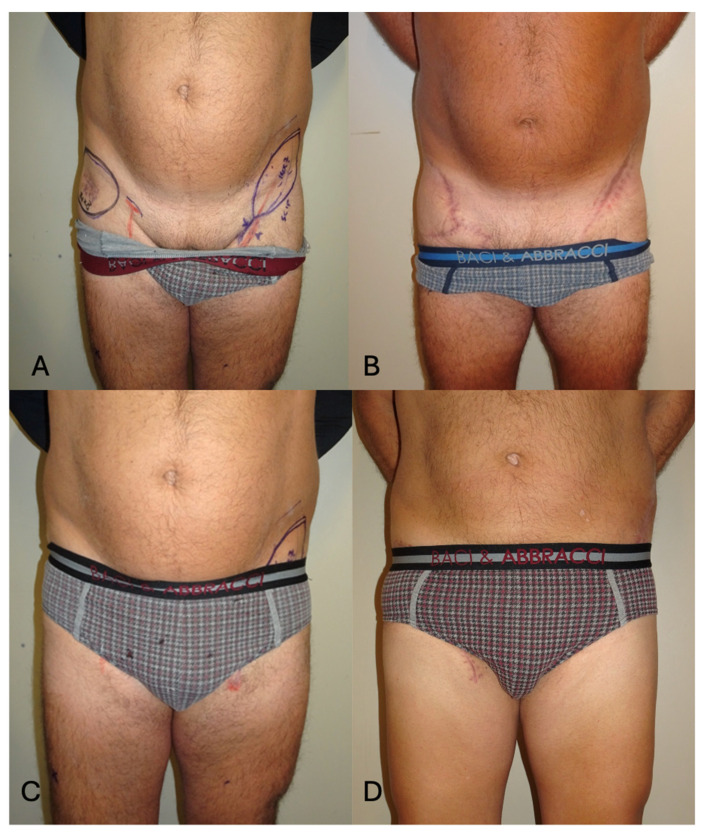
(**A**) Pre-operative frontal view with SCIP flap planning. (**B**) Six-month post-operative view showing a well-integrated flap allowing a like-for-like reconstruction with tissues of a similar color and texture from the contralateral side. The contour was restored, and the donor site was closed primarily in an aesthetic fashion. (**C**) Pre-operative frontal view with patient wearing underwear. (**D**) Six-month post-operative view with underwear, showing how the aesthetic approach employed allowed to us hide both the donor and recipient sites in well-concealed anatomical regions. The patient was very satisfied with the aesthetic result of the reconstruction.

**Figure 6:**
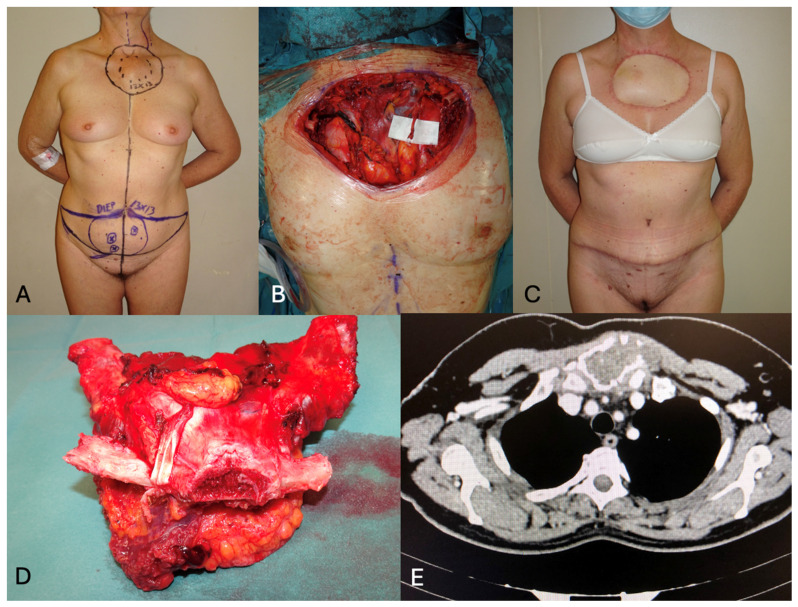
(**A**) a case of chondrosarcoma involving the sternal manubrium and the first 2 ribs bilaterally in a 60-year-old female patient. The extensive defect measured 13 × 13 cm. The patient was scheduled for DIEP flap reconstruction, which was the only available donor site to allow primary closure in an aesthetically acceptable fashion in the supine operative position. (**B**) Intra-operative view of the extensive defect with exposure of both the lungs and aortic arch. The left internal mammary vessels were prepared for anastomoses. (**C**) Six-month post-operative view showing a well-settled flap with aesthetic improvement of the abdominal contour after closure in an abdominoplasty fashion. (**D**) Excised tumor. (**E**) Pre-operative CT showing the degree of local infiltration.

This drawback pushed us to find alternative solutions, leading us to increasingly use lower abdominal free perforator flaps, such as the SCIP flap for moderate-to-wide-sized defects (Figure 4 and Figure 5) and the DIEP flap for extensive soft-tissue defects (Figure 6).

**Figure 7:**
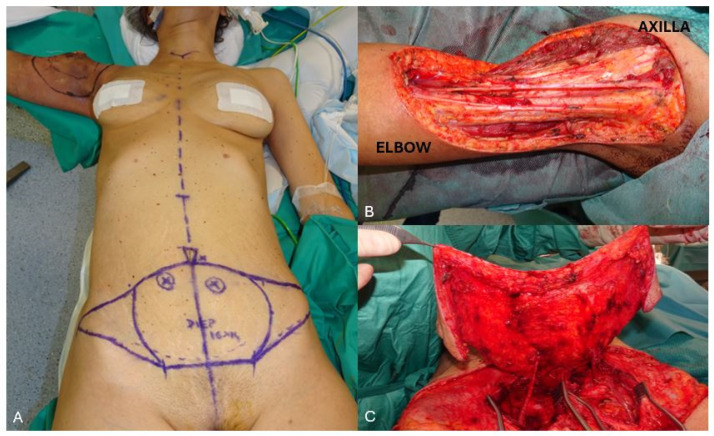
(**A**) a young female patient presented with an irradiated recurrent undifferentiated sarcoma involving the proximal right upper arm. The defect was almost completely circumferential, measuring 16 × 14 cm. The patient was scheduled for DIEP flap reconstruction, which was the only available donor site to allow primary closure in an aesthetically acceptable fashion in the supine operative position. (**B**) Intra-operative view of the extensive defect with exposure of the major neuro-vascular structures. (**C**) The patient was very thin and narrow waisted and the defect quite extensive. To safely cover the entire defect, all the lower abdominal tissue was required. Therefore, a bipedicled bilateral DIEP flap was harvested.

**Figure 8:**
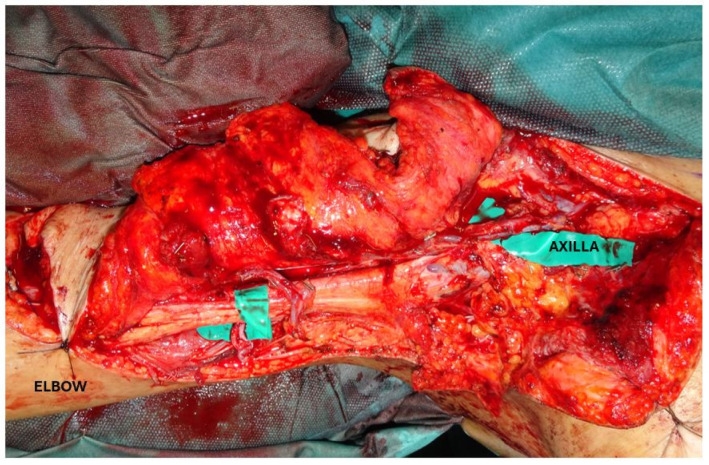
Intra-operative view of the flap after revascularization. Two sets of end-to-end anastomoses were performed, with one pedicle anastomosed to the thoracoacromial vessels, which were rerouted from the upper trunk to the proximal part of the defect, and the other pedicle was anastomosed with a brachial artery recurrent branch, located in the distal part of the defect. The use of both DIEP pedicles allowed us to transfer the entire abdominal pannus with 100% flap survival.

**Figure 9:**
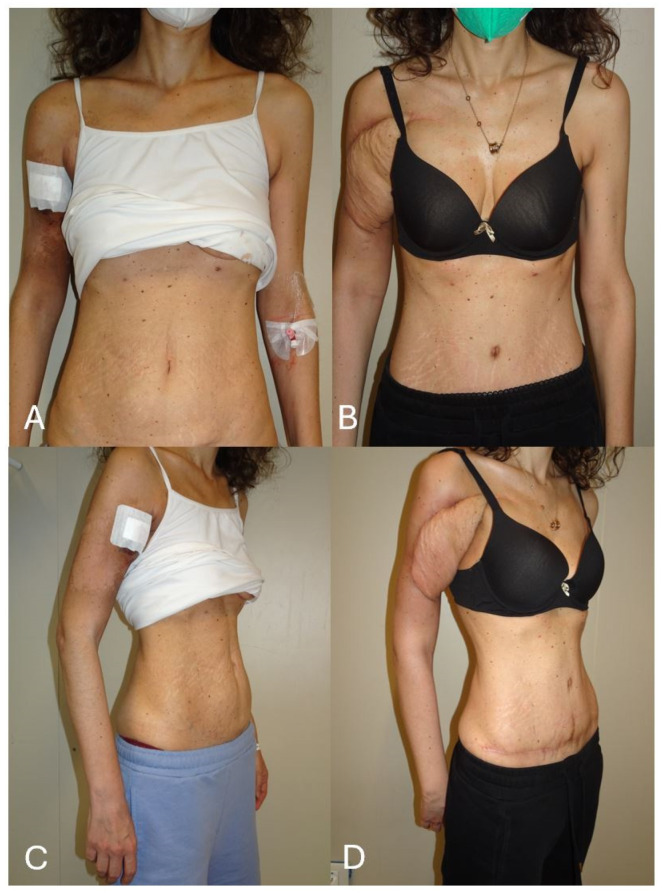
(**A**) Pre-operative frontal view. (**B**) Six-month post-operative frontal view showing a well-settled flap with aesthetic improvement of the abdominal contour after closure in an abdominoplasty fashion. The patient was very happy with the aesthetic strategy adopted for reconstructing such a complex defect. (**C**) Pre-operative three-quarters view. (**D**) Six-month post-operative three-quarters view. Noteworthy is the improvement of the abdominal silhouette.

The DIEP flap, first described by Koshima and Soeda in 1989 [17], has become the gold standard in autologous breast reconstruction [18] due to its ability to provide a good amount of tissue while preserving the rectus abdominis muscle. Meanwhile, its employment for reconstructing other anatomical regions is infrequent [19] and typically not prioritized as a primary option in alternative reconstructive scenarios. One of its main advantages is the possibility to harvest it as an extended, pre-expanded, bipedicled flap (Figure 7, Figure 8 and Figure 9), making it one of—if not the—largest flap in the human body [20,21,22]. The DIEP flap also offers advantages considering its vascular anatomy. Particularly, when dealing with reconstructions in regions with suboptimal vascularity, such as in lower limb cases, the perfusion of this flap can be augmented through supercharging and/or superdraining techniques via the superficial circumflex iliac system [23]. Unlike other skin paddle designs that have been described in the literature (oblique [24], vertical [25], “kiss design” [26]), this flap is normally harvested in a horizontal fashion, allowing for a more aesthetic closure of the donor site. In a retrospective analysis conducted by Abdelfattah et al. [27], encompassing 563 flaps utilized in lower limb reconstruction, the findings revealed that the DIEP flap stands out for its considerable size (836.2 ± 210.3 cm^2^), longest pedicle length (11.7 ± 1.4 cm), and notable thickness (11.1 ± 3.9 mm) compared to other flap options.

Several other extra-mammary applications of the DIEP flap have been described in the literature, including its use for thigh reconstructions, where it can be maintained as a pedicled [28,29] flap or set up as a free flap [30], and for upper limb reconstructions when traditional local flaps are insufficient for the reconstruction of large defects [31,32]. Regarding the upper limbs, the DIEP flap is a good indication for the reconstruction of large and proximal defects, while thinner and more pliable flaps are preferred for more distal reconstructions [33].

A second alternative “aesthetic” option to treat moderate-to-wide-sized defects in our experience was the SCIP flap [34], which offered a viable alternative for defects in the lower extremities [35], perineum, and abdomen. This is the thinnest skin flap presently available and can be used for moderate-sized defects [36]. It can be harvested in a chimeric fashion including the sartorius muscle [37] and/or the iliac bone [38,39]. According to Yoshimatsu and colleagues [40], the SCIP flap has a high success rate in sarcoma reconstruction when compared to other flaps such as the ALT and latissimus dorsi (LD) because of its higher degree of freedom with which the recipient vessel can be chosen. Because the diameter of the SCIA is relatively small (usually around 1.0 mm), it can be anastomosed in an end-to-end or an end-to-side fashion to the source arteries in any chosen region, especially for upper and lower extremity defects, where finding an adequate recipient vessel can sometimes be challenging.

Moreover, the SCIP flap harvest, like the DIEP flap, offers the advantages of low donor-site morbidity, an inconspicuous scar that can be easily hidden in the inguinal crease, and minimal functional morbidity, thereby reducing the impact of surgery on patients undergoing this kind of reconstruction.

Unlike other perforator flaps such as the ALT flap, the thoracodorsal artery perforator flap, the profunda femoris artery perforator flap, and the medial sural artery perforator flap, the SCIP donor site can still be closed directly even with a skin paddle width exceeding 10–12 cm, and up to 14 cm with the hip flexed [40].

In comparison to larger flaps such as the DIEP and LD flaps, the SCIP flap has dimensional limitations. However, in cases of large defects, the literature describes the possibility of combining the SCIP flap with either the superficial inferior epigastric artery flap or the deep inferior epigastric artery perforator flap to cover extensive soft-tissue defects, especially in the extremities and trunk [41]. However, the SCIP flap’s main limitation lies in its anatomical variations, relatively short pedicle length, and thinness. As a result, it is not advisable for inexperienced microsurgeons, especially if dealing with deep wounds or cases where a longer pedicle is necessary.

Having said the above, our study was not aimed at demonstrating the superiority of one flap over another in an absolute sense. Our goal was to define a process for choosing the most appropriate flap considering both the defect to be reconstructed and the donor site.

Generally speaking, the dimensional criterion is the first to be considered; indeed, ALT, SCIP, and DIEP flaps can be used for defects of progressively increasing size (8–10 cm, 10–14 cm, 14–16 cm, and more), always trying to reduce donor site morbidity and achieve its primary closure. In this scenario, the DIEP flap represents the most versatile option, allowing us to resurface big and extremely extensive defects in its single pedicle or bilateral pedicle conjoined versions, respectively. Moreover, as previously stated, the DIEP flap can be combined with other adjacent pedicles (SCIA/v-, SIEA/v, DCIA/v, LAPs) to extend the possibility of coverage to the most complex defect scenarios.

Nevertheless, the dimensional criterion should be weighed against other factors, such as the need to operate in a certain anatomical position: for example, SCIP and DIEP flaps can only be harvested in the supine position and are better indicated for defects located on the anterior body surface. Meanwhile, for forced lateral or prone position resections, other flaps should be used to reduce operative times and maintain surgical efficiency, such as LD/TDAP flaps [5,6] and ALT/PAP [42] flaps for large and moderate-sized defects, respectively.

The depth of the defect is another factor to take into account; the ALT flap and its thin variants described in the literature, along with the SCIP flap, are generally preferable for reconstructing shallow substance losses, such as those at the extremities or at joint junctions, where a thick flap would result in inappropriate aesthetic outcomes and limited mobility. When facing defects involving deep structures and leading to big cavities, especially in irradiated fields, the reconstructive surgeon will need a thicker flap, such as the myocutaneous ALT flap harvested with the underlying vastus lateralis muscle, or the DIEP flap.

In our opinion, in the realm of reconstructive surgery for sarcomas, the selection of a free flap must extend beyond considerations solely focused on the recipient site and surrounding recipient vessels [43]. Indeed, the consequences at the donor site warrant equal attention, as the resulting scar can significantly influence the patient’s overall well-being and recovery journey. Opting for flaps sourced from regions where aesthetic restoration is feasible not only improves the patient’s quality of life but also promotes their physical and psychological rehabilitation. In this context, the utilization of Patient-Reported Outcome Measures (PROMs) emerges as a pivotal tool. These measures provide invaluable insights into the patient experience, allowing us to comprehensively evaluate the impact of a chosen reconstructive approach. Specifically, instruments like the SCAR-Q questionnaire enable a thorough assessment of various facets, including scar appearance, associated symptoms, and its psychosocial impact.

Primary closure of the thigh, if performed carefully, can result in an aesthetically acceptable appearance, but often does not meet the standards of aesthetic and scar softness achieved with closure of donor sites from abdominal flaps such as the DIEP and SCIP/SIEA [44,45]. Even less aesthetically acceptable are ALT closures whose dimensions do not allow for primary closure, thus requiring the use of skin grafts. Conversely, scars resulting from the closure of abdominal flap donor sites are typically hidden beneath clothing lines and may be thinner, softer, and less prominent.

In patients with comparable BMIs, and therefore similar subcutaneous thicknesses, allowing for free selection between ALT, SCIP, and DIEP flaps, our study focused on the preference for abdominal flaps over ALT. This preference was based on the aesthetic restoration of the donor site, which we assessed using the SCAR-Q questionnaire for patient satisfaction. The results show that patient satisfaction with the donor site measured using the SCAR-Q questionnaire was significantly higher in the DIEP/SCIP group when compared with the ALT group (*p* < 0.001), especially in the appearance scale, indicating a superiority of the lower abdominal area as an aesthetic donor site.

## 5. Conclusions

Lower abdominal perforator free flaps, like DIEP and SCIP flaps, are a versatile option for use to reconstruct large soft-tissue defects following sarcoma resection in the supine position, allowing for a higher patient satisfaction with the donor site, which is a feature worth considering when planning a reconstructive procedure.

## Figures and Tables

**Table 1:** Patient demographic characteristics and operative variables. The bold numbers indicate statistically significant values.

	Abdominal flaps (DIEP, SCIP) (%) *n* = 22	Thigh flaps (ALT, TFL, AMT) (%) *n* = 18	*p*-Value
**Patient demographics**			
Gender			
Male	6 (27.3%)	14 (77.8%)	**0.013**
Female	16 (72.7%)	4 (22.2%)	**0.013**
Mean age, y (range)	58.9 (range 33–88)	58.9 (range 19–81)	0.994
Body Mass Index, kg/cm^2^ (range)	24.9 (range 17.6–34)	26 (range 20.5–36.3)	0.355
Active smoker			
Yes	4 (18.2%)	4 (22.2%)	>0.999
No	18 (81.8%)	14 (77.8%)	>0.999
Mean length of follow-up, months (range)	15.3 (range 6–36)	21.3 (range 6–41)	0.079
**Perioperative treatments**			
Neoadjuvant RT			
Yes	7 (31.8%)	4 (22.2%)	>0.999
No	15 (68.2%)	14 (77.8%)	>0.999
Neoadjuvant CT			
Yes	7 (31.8%)	2 (11.1%)	0.789
No	15 (68.2%)	16 (88.9%)	0.789
Adjuvant RT			
Yes	3 (13.6%)	9 (50%)	0.146
No	19 (86.4%)	9 (50%)	0.146
Adjuvant CT			
Yes	0 (0%)	3 (16.7%)	0.946
No	22 (100%)	15 (83.3%)	0.946
**Operative variables**			
Type of flap	SCIP = 7 (31.8%) DIEP = 15 (68.2%)	ALT = 15 (83.3%) TFL = 2 (11.1%) AMT = 1 (5.6%)	-
Defect size cm^2^ (range)	292 (range 150–540)	247 (range 108–600)	0.123
Flap size cm^2^, (range)	282 (range 150–540)	264 (range 165–600)	0.607
Operative time, min (range)	655.2 (range 330–918)	527 (range 318–706)	**0.003**
Skin grafting of donor site	0 (0%)	10 (55.5%)	**0.002**
Healing time, days (range)	31.3 (range 15–75)	80 (range 21–160)	**0.0005**
**Recipient site complications**			
No	17 (77.3%)	15 (83.3%)	>0.999
Yes	5 (22.7%)	3 (16.7%)	>0.999
Partial flap loss	1 (20%)	0 (0%)	
Total flap loss	1 (20%)	1 (33.3%)	
Seroma	3 (60%)	2 (66.7%)	
**Donor site complications**			
No	19 (86.4%)	9 (50%)	**0.017**
Yes (infection, seroma)	3 (13.6%)	9 (50%)	**0.017**

**Table 2:** Post-operative SCAR-Q scores.

	Abdominal Flaps *n* = 22	Thigh Flaps *n* = 18	Paired *t*-Test (*p*-Value)
Appearance scale	63.3 ± 12.5	46.6 ± 9.82	**<0.0001**
Symptom scale	60.5 ± 9.46	42.9 ± 9.17	**<0.0001**
Psychosocial impact	57.2 ± 12.1	45.0 ± 8.57	**0.007**

## Data Availability

Data supporting the findings of this study are available upon request to the authors.

## References

[1] Penna V., Iblher N., Momeni A., Stark G.B., Bannasch H. (2011). Free tissue transfer in reconstruction following soft tissue sarcoma resection. Microsurgery.

[2] Elswick S.M., Wu P., Arkhavan A.A., Molinar V.E., Mohan A.T., Sim F.H., Martinez-Jorge J., Saint-Cyr M. (2019). A reconstructive algorithm after thigh soft tissue sarcoma resection including predictors of free flap reconstruction. J. Plast. Reconstr. Aesthet. Surg..

[3] Brunetti B., Salzillo R., De Bernardis R., Tenna S., Camilloni C., Persichetti P. (2024). Conjoined thoracodorsal perforator-supercharged dorsal intercostal artery perforator propeller flap for reconstruction of a complex upper back defect: Case report and review of the literature on supercharged pedicled perforator flaps. Microsurgery.

[4] Marré D., Buendía J., Hontanilla B. (2012). Complications following reconstruction of soft-tissue sarcoma: Importance of early participation of the plastic surgeon. Ann. Plast. Surg..

[5] Zhang Y.X., Hayakawa T.J., Levin L.S., Hallock G.G., Lazzeri D. (2016). The Economy in Autologous Tissue Transfer: Part 1. The Kiss Flap Technique. Plast. Reconstr. Surg..

[6] Brunetti B., Salzillo R., Tenna S., Cagli B., Coppola M.M., Petrucci V., Camilloni C., Zhang Y.X., Persichetti P. (2022). Total autologous breast reconstruction with the Kiss Latissimus Dorsi Flap. J. Plast. Reconstr. Aesthetic Surg. JPRAS.

[7] Brunetti B., Salzillo R., Tenna S., Petrucci V., Morelli Coppola M., Valeri S., Persichetti P. (2023). Abdominal wall reconstruction with the free functional L-shaped latissimus dorsi flap: A case report. Microsurgery.

[8] Sharma K., Steele K., Birks M., Jones G., Miller G. (2019). Patient-Reported Outcome Measures in Plastic Surgery: An Introduction and Review of Clinical Applications. Ann. Plast. Surg..

[9] Klassen A.F., Ziolkowski N., Mundy L.R., Miller H.C., McIlvride A., DiLaura A., Fish J.M., Pusic A.L.M. (2018). Development of a New Patient-reported Outcome Instrument to Evaluate Treatments for Scars: The SCAR-Q. Plast. Reconstr. Surg. Glob. Open.

[10] Salzillo R., Barone M., Persichetti P. (2023). Does a High-Quality Scar Overcome its Length? Italian Validation of the SCAR-Q Questionnaire. Aesthetic Plast. Surg..

[11] Chen H.c., Tang Y.b. (2003). Anterolateral thigh flap: An ideal soft tissue flap. Clin. Plast. Surg..

[12] Miyamoto S., Kageyama D., Arikawa M., Kagaya Y., Fukunaga Y. (2021). Combined use of ipsilateral latissimus dorsi flap and anterolateral thigh flap to reconstruct extensive trunk defects. Microsurgery.

[13] Brunetti B., Morelli Coppola M., Tenna S., Salzillo R., Petrucci V., Pazzaglia M., Valeri S., Alloni R., Vincenzi B., Tonini G. (2024). Thigh reconstruction between form and function: An algorithm for flap selection based on a series of 70 oncological patients. Microsurgery.

[14] Moschella F., D’Arpa S., Pirrello R., Cordova A. (2010). Posterior compartment of the lower leg reconstruction with free functional rectus femoris transfer after sarcoma resection. J. Plast. Reconstr. Aesthetic Surg. JPRAS.

[15] Miyamoto S., Kagaya Y., Arikawa M., Kobayashi E. (2016). Combined Use of an Anterolateral Thigh Flap and Superficial Inferior Epigastric Artery Flap for Reconstruction of an Extensive Abdominal Wall Defect. Plast. Reconstr. Surg. Glob. Open.

[16] Collins J., Ayeni O., Thoma A. (2012). A systematic review of anterolateral thigh flap donor site morbidity. Can. J. Plast. Surg. J. Can. Chir. Plast..

[17] Koshima I., Soeda S. (1989). Inferior epigastric artery skin flaps without rectus abdominis muscle. Br. J. Plast. Surg..

[18] Blondeel P.N. (1999). One hundred free DIEP flap breast reconstructions: A personal experience. Br. J. Plast. Surg..

[19] Guinier C., de Clermont-Tonnerre E., Tay J.Q., Ng Z.Y., Cetrulo C.L., Lellouch A.G. (2023). The deep inferior epigastric artery perforator flap: A narrative review on its various uses in non-breast reconstruction. Ann. Transl. Med..

[20] Mahajan A.L., Van Waes C., D’Arpa S., Van Landuyt K., Blondeel P.N., Monstrey S., Stillaert F.B. (2016). Bipedicled DIEAP flaps for reconstruction of limb soft tissue defects in male patients. J. Plast. Reconstr. Aesthetic Surg. JPRAS.

[21] Melnikov D.V., Starceva O.I., Ivanov S.I., Garmi R., Sinelnikov M.Y. (2020). A Unique Case of Lower Limb Soft Tissue Reconstruction with a Prefabricated Bipedicled Deep Inferior Epigastric Artery Flap. Plast. Reconstr. Surg. Glob. Open.

[22] Grinsell D., Saravolac V., Rozen W.M., Whitaker I.S. (2012). Pre-expanded bipedicled deep inferior epigastric artery perforator (DIEP) flap for paediatric lower limb reconstruction. J. Plast. Reconstr. Aesthetic Surg. JPRAS.

[23] Qing L.M., Tang J.Y. (2019). Use of intraflap and extraflap microvascular anastomoses in combination for facilitating bipedicled DIEP/SIEA free flap for reconstruction of circumference soft tissue defect of extremity. Microsurgery.

[24] Van Landuyt K., Blondeel P., Hamdi M., Tonnard P., Verpaele A., Monstrey S. (2005). The versatile DIEP flap: Its use in lower extremity reconstruction. Br. J. Plast. Surg..

[25] Akdag O., Karamese M., Yıldıran G.U., Sutcu M., Tosun Z. (2018). Foot and ankle reconstruction with vertically designed deep inferior epigastric perforator flap. Microsurgery.

[26] Luo Z., Qing L., Zhou Z., Wu P., Yu F., Tang J. (2019). Reconstruction of Large Soft Tissue Defects of the Extremities in Children Using the Kiss Deep Inferior Epigastric Artery Perforator Flap to Achieve Primary Closure of Donor Site. Ann. Plast. Surg..

[27] Abdelfattah U., Power H.A., Song S., Min K., Suh H.P., Hong J.P. (2019). Algorithm for Free Perforator Flap Selection in Lower Extremity Reconstruction Based on 563 Cases. Plast. Reconstr. Surg..

[28] Scaglioni M.F., Giunta G., Barth A.A., Giovanoli P. (2020). A pedicled split extended vertical deep inferior epigastric (s-vDIEP) flap and an adipo-dermal thigh local flap for the reconstruction of the medial thigh compartment after sarcoma resection: A case report. Microsurgery.

[29] Fernández Garrido M., Pereira N., López Fernández S., Vega C., Masià J. (2018). Turbocharged bilateral pedicled DIEP flap for reconstruction of thigh defect without recipient vessels: A case report. Microsurgery.

[30] Bota O., Spindler N., Sauber J., Aydogan E., Langer S. (2017). Double-Pedicled Free Deep Inferior Epigastric Perforator Flap for the Coverage of Thigh Soft-Tissue Defect. Plast. Reconstr. Surg. Glob. Open.

[31] Chen H.C., Tang Y.B., Mardini S., Tsai B.W. (2004). Reconstruction of the hand and upper limb with free flaps based on musculocutaneous perforators. Microsurgery.

[32] Hamdi M., Van Landuyt K., Monstrey S., Blondeel P. (2004). A clinical experience with perforator flaps in the coverage of extensive defects of the upper extremity. Plast. Reconstr. Surg..

[33] Wang H.D., Alonso-Escalante J.C., Cho B.H., DeJesus R.A. (2017). Versatility of Free Cutaneous Flaps for Upper Extremity Soft Tissue Reconstruction. J. Hand Microsurg..

[34] Koshima I., Nanba Y., Tsutsui T., Takahashi Y., Urushibara K., Inagawa K., Hamasaki T., Moriguchi T. (2004). Superficial circumflex iliac artery perforator flap for reconstruction of limb defects. Plast. Reconstr. Surg..

[35] Hong J.P., Sun S.H., Ben-Nakhi M. (2013). Modified superficial circumflex iliac artery perforator flap and supermicrosurgery technique for lower extremity reconstruction: A new approach for moderate-sized defects. Ann. Plast. Surg..

[36] Goh T.L.H., Park S.W., Cho J.Y., Choi J.W., Hong J.P. (2015). The search for the ideal thin skin flap: Superficial circumflex iliac artery perforator flap—A review of 210 cases. Plast. Reconstr. Surg..

[37] Yoshimatsu H., Yamamoto T., Hayashi N., Kato M., Iida T., Koshima I. (2017). Reconstruction of the ankle complex wound with a fabricated superficial circumflex iliac artery chimeric flap including the sartorius muscle: A case report. Microsurgery.

[38] Yoshimatsu H., Iida T., Yamamoto T., Hayashi A. (2018). Superficial Circumflex Iliac Artery-Based Iliac Bone Flap Transfer for Reconstruction of Bony Defects. J. Reconstr. Microsurg..

[39] Iida T., Narushima M., Yoshimatsu H., Yamamoto T., Araki J., Koshima I. (2013). A free vascularised iliac bone flap based on superficial circumflex iliac perforators for head and neck reconstruction. J. Plast. Reconstr. Aesthetic Surg. JPRAS.

[40] Yoshimatsu H., Karakawa R., Fuse Y., Tanakura K., Yamamoto T., Okada A., Daniel B.W., Yano T. (2021). Use of the superficial circumflex iliac artery perforator flap for reconstruction after sarcoma resection. J. Surg. Oncol..

[41] Yoshimatsu H., Hayashi A., Karakawa R., Yano T. (2020). Combining the superficial circumflex iliac artery perforator flap with the superficial inferior epigastric artery flap or the deep inferior epigastric artery perforator flap for coverage of large soft tissue defects in the extremities and the trunk. Microsurgery.

[42] Karakawa R., Yoshimatsu H., Maeda E., Shibata T., Tanakura K., Kuramoto Y., Miyashita H., Yano T. (2020). Use of the Profunda Femoris Artery Perforator Flap for Reconstruction after Sarcoma Resection. Plast. Reconstr. Surg. Glob. Open.

[43] Brunetti B., Petrucci V., Tenna S., Morelli Coppola M., Salzillo R., Putti A., Camilloni C., Pazzaglia M., Persichetti P. (2024). «Extra-anatomical Pedicle Rerouting» An alternative technique to obtain new recipient vessels for microsurgical reconstruction in unfavorable clinical situations. J. Plast. Reconstr. Aesthetic Surg. JPRAS.

[44] Miyamoto S., Arikawa M., Kagaya Y. (2020). The use of lower abdominal perforator flaps in soft-tissue reconstruction after sarcoma resection. Microsurgery.

[45] Franchi A., Patanè L., Hummel C.H., Jung F. (2024). Confusion Regarding the Anatomy of the Superficial Inferior Epigastric Artery and the Superficial Circumflex Iliac Artery Superficial Branch. Plast. Reconstr. Surg. Glob. Open.

